# Serosurvey of Immunity to Monkeypox (Mpox) Virus Antigens in People Living with HIV in South Florida

**DOI:** 10.3390/pathogens12111355

**Published:** 2023-11-15

**Authors:** Jonah Kupritz, Savita Pahwa, Suresh Pallikkuth

**Affiliations:** Department of Microbiology and Immunology, University of Miami Miller School of Medicine, Miami, FL 33136, USA; jkupritz@med.miami.edu (J.K.); spahwa@med.miami.edu (S.P.)

**Keywords:** mpox, monkeypox, vaccine, diagnostic, seroprevalence, serosurveillance, immunoassay

## Abstract

Mpox is an infectious disease caused by the monkeypox virus (MPXV) belonging to the Orthopoxvirus (OPXV) genus, which includes smallpox and vaccinia virus (VACV). A global mpox outbreak which began in May 2022 has infected more than 88,000 people. VACV-based vaccines provide protection against mpox disease but complicate the use of serological assays for disease surveillance. We tested the reactivity of serum IgG from Modified Vaccinia Ankara-Bavarian Nordic (MVA-BN)-vaccinated (*n* = 12) and convalescent mpox-infected (*n* = 5) individuals and uninfected, non-vaccinated controls (*n* = 32) to MPXV/VACV proteins A27, A29, A30, A35, B16, B21, C19, D6, E8, H3, I1, and L1. Using a subset of MPXV antigen-based assays (A35, B16, E8, H3, and I1), we conducted a mpox antibody survey of serum from 214 individuals, including 117 (54.7%) people with HIV (PWH) collected between June 2022 and January 2023, excluding individuals who reported recent mpox vaccination or infection, and 32 young, pre-pandemic controls. The convalescent sera reacted strongly to most tested antigens. Vaccine sera responses were limited to A35, E8, H3, and I1. IgG antibody to E8 was markedly elevated in all vaccinated individuals. B16 IgG showed high sensitivity (100% [95% CI: 56.55–100.0%]) and specificity (91.67% [64.61–99.57%]) for distinguishing infection from MVA-BN vaccination, while E8 IgG showed 100% [75.75–100] sensitivity and 100% [79.61–100] specificity for detecting and distinguishing vaccinated individuals from controls. We identified 11/214 (5.1%) recent serum samples and 1/32 (3.1%) young, pre-pandemic controls that were seropositive for ≥2 MPXV antibodies, including 6.8% of PWH. Seropositivity was 10/129 (7.8%) among males compared to 1/85 (1.2%) among females. Our findings provide insight into the humoral immune response to mpox and demonstrate the usefulness of inexpensive, antigen-based serosurveillance in identifying asymptomatic or unreported infections.

## 1. Introduction

Mpox (formerly known as monkeypox) is a disease caused by the monkeypox virus (MPXV). MPXV shares high structural homology with other Orthopoxvirus (OPXV) genus members, including variola virus and vaccinia virus (VACV) [1,2]. Since the onset of the global outbreak in May 2022, more than 85,000 confirmed mpox cases have been registered in locations that have not historically reported mpox [3,4], including more than 1600 mpox cases in South Florida, where HIV incidence is high [5,6]. In South Florida’s two largest counties, Miami-Dade and Broward, the prevalence of people with HIV (PWH) exceeds 1% of the total population, with nearly 1700 new diagnoses in 2022 [7]. Up to half of mpox cases have been found among PWH, who are at higher risk of developing more severe forms of the disease [8,9]. Mpox cases have primarily been identified via passive surveillance mechanisms, whereby symptomatic individuals present to healthcare providers who report positive or suspected cases to health jurisdictions [10,11]. Passive surveillance is relatively low-cost and useful for monitoring case trends longitudinally but has low sensitivity and may provide demographically unreliable data; additionally, it is unlikely to capture asymptomatic mpox, which comprises a small portion of infections [12]. To date, few studies have been conducted measuring the presence of mpox antibodies in large populations (seroprevalence), which may better estimate the true infection rates [13,14,15]. Mpox serology typically uses purified VACV particle-based ELISA, which is limited by access to VACV culture and immunological cross-reactivity from previous vaccinations [16]. Individuals may have OPXV immunity from live attenuated VACV-based vaccines administered as routine smallpox prophylaxis prior to 1980 or during the ongoing mpox outbreak [17,18]. Currently, Bavarian Nordic’s MVA-BN vaccine based on a live attenuated VACV strain, Modified Vaccinia Ankara (MVA), is the main vaccine being used for the prevention of mpox in the United States [19]. MVA-BN vaccination has proven highly safe and effective (>85%) at preventing mpox disease after two doses, although breakthrough cases have emerged [20,21,22,23]. A small number of studies have demonstrated the usefulness of peptide-based assays in the detection of vaccine- and infection-derived mpox immunity, with high sensitivities and specificities achieved using peptide conjugates or single proteins, such as B21, which is present in MPXV but absent in VACV [24,25,26,27]. Mpox infection has been found to elicit a strong antibody response to multiple Orthopoxvirus antigens [28]. In contrast, low levels of MPXV-neutralizing antibodies have been found after vaccination with MVA-BN, which may offer protection via T-cell or complement-dependent mechanisms [29,30,31]. Currently, several recombinant MPXV proteins and their VACV homologs are commercially available at a low cost, including secreted (B16, D6) intracellular mature virus (IMV) (A29, A30 E8, H3, L1), extracellular enveloped virus (EEV) (A35, C19), and DNA-binding core protein (I1L) [32]. Other proteins, such as the large membrane-associated glycoprotein B21, require custom production. We sought to determine the humoral immune response to OPXV antigens after mpox infection and MVA-BN vaccination and develop antigen-based assays for the detection of mpox- and vaccine-derived immunity. We used these assays to measure the prevalence of mpox IgG antibodies in a large number of serum samples from PWH and PWoH in South Florida collected during the global outbreak in order to better understand the true infection rates and estimate the prevalence of mpox immunity at the community level.

## 2. Materials and Methods

The vaccine and control sera were obtained with IRB approval from two ongoing studies at the University of Miami Miller School of Medicine in Miami, Florida: OPIS (Opioid Immunity Study; R01DA051202; NCT04304768) and Tfh Dysfunction in HIV and Aging (R01AG068110; NCT04487041). Both studies use the annual influenza vaccine as a probe to evaluate immune dysfunction in virally suppressed people with HIV (PWH) and people without HIV (PWoH). OPIS enrolls opioid-using and non-using PWH and PWoH aged 18–60 years old; the Aging study enrolls equal numbers of PWH and PWoH aged 18–40 or >65 years old. All PWH participants in both studies were virally suppressed on ART. The mpox sera were obtained from an IRB-approved sample repository (Cantor BioConnect, Santee, CA). Participants were surveyed about past mpox vaccination and history of confirmed mpox infection. Mpox-vaccinated individuals (*n* = 12) received 1–2 doses of Modified Vaccinia Ankara-Bavarian Nordic (MVA-BN) vaccine ≥2 weeks prior to serum collection and had no history of symptomatic mpox infection. The average age of vaccinated individuals was 49 years (range = 23–66, SD = 15.4); 9/12 (75%) were PWH, and 10/12 (83.3%) were male. The mpox sera (*n* = 5) were from individuals who recovered from PCR-confirmed mpox. The average age of mpox-convalescent individuals was 32.2 years (range = 28–39, SD = 4.8), 5/5 (100%) were male, and one individual had received a dose of the MVA-BN vaccine. All five mpox-convalescent individuals were HIV-seronegative (Table 1). The control sera (*n* = 32) were collected prior to the global mpox outbreak from PWoH less than 40 years of age (born after routine smallpox vaccination ended).

As a preliminary screening, indirect IgG ELISAs were performed on a small subset of the sera (*n* = 5 convalescent mpox patients, *n* = 3 MVA-BN-vaccinated, and *n* = 3 uninfected/unvaccinated controls) using commercially available antigens from MPXV (A29, A30, A35, B16, C19, D6, E8, H3, I1, L1) (RayBiotech, Peachtree Corners, GA, USA) and VACV (A27, an ortholog of MPXV A29) (Sino Biological US Inc., Wayne, PA, USA). We also tested a custom-synthesized full-length MPXV protein: B21 (Creative Diagnostics, Shirley, NY, USA). Antigens with high reactivity among convalescent or vaccine sera and low reactivity among controls were validated among additional vaccinated and control samples (in total: *n* = 5 convalescent, *n* = 12 vaccinated, *n* = 15 controls). On each plate, an 8-point standard curve was run in duplicate with serial two-fold diluted pooled serum from the convalescent mpox samples in addition to a blank (sample buffer). Briefly, the ELISAs were prepared by plating 2 µg/mL of antigen in phosphate-buffered saline (PBS) overnight at 4 °C. Between each step, the plates were washed with 0.1% Tween 20 in PBS. The sera and antibodies were diluted in sample buffer (1% nonfat dry milk in PBS). Following 1 h of blocking at room temperature with 3% nonfat dry milk in PBS with 0.1% Tween 20, the sera were added at 1:100 (E8 assay) or 1:50 (all other assays) and incubated for 1 h at 37 °C. An HRP-conjugated goat anti-human IgG secondary antibody (Thermo Fisher, Waltham, MA, USA) was added at 1:6000 (E8 assay) or 1:4000 (all other assays) for 1 h at room temperature, followed by 10 min development with TMB at room temperature. Development was stopped using sulfuric acid and the ODs were read at 450 nm. Arbitrary units (AU) were interpolated from the ODs based on the standard curves generated using the pooled mpox-convalescent sera. The highest standard, run at a 1:50 dilution, was set to a final concentration of 5000 AU/mL. Seroprevalence was assessed using the validated antigen assays (A35, B16, E8, H3, I1) in samples from 214 individuals (60.3% male; 54.7% PWH; average age 48.7 years, standard deviation (SD) = 14.7 years) collected between June 2022 and January 2023 who denied a history of mpox vaccination or infection in the self-report questionnaire (Supplementary File S1). On each plate, the full set of control sera (*n* = 32) was run. The antibody positivity cutoffs were set at two and a half standard deviations above the negative control mean. Due to some level of background reactivity in the controls, the mpox seropositivity was defined as seropositivity for two or more antibodies. 

Two-tailed Mann–Whitney or Kruskal–Wallis tests with Dunn’s multiple comparison tests were performed for the two- or multiple-group comparisons, respectively. For the sensitivity and specificity of individual assays, receiver operating characteristic (ROC) curves were generated; 95% confidence intervals were calculated using the Wilson/Brown method. Specificity for vaccination was calculated using uninfected, unvaccinated individuals as the controls; for specificity of infection, vaccinated individuals were used as the controls. Associations between mpox seropositivity and demographic data (HIV status, age, and gender) were determined using chi-square and Fisher’s exact testing. Multinomial logistic regression analysis was performed to address the confounding effect of HIV status, gender, and age on the antibody responses. The threshold for statistical significance was set at *p* ≤ 0.05. Statistical analyses and graphing were performed using GraphPad Prism 9.0. Detailed ELISA-related information is available in Supplementary File S2.

## 3. Results

### 3.1. Preliminary Screen

**Figure 1:**
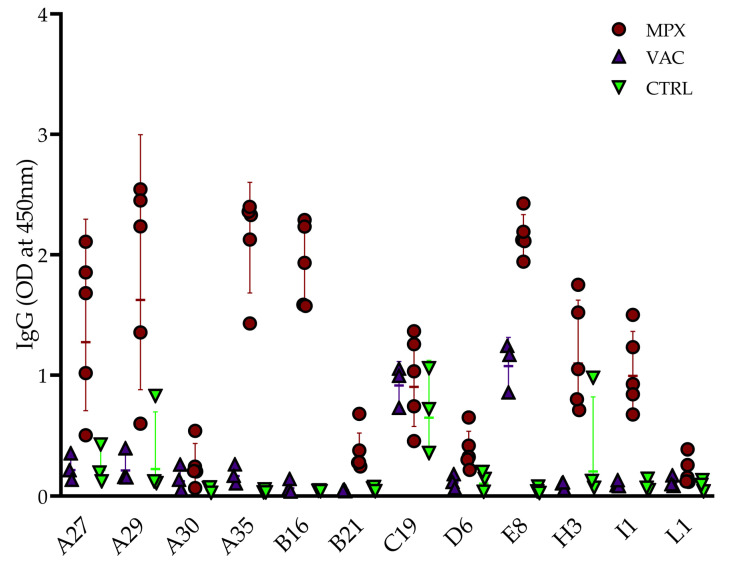
Preliminary screen of serum IgG reactivity to MPXV/VACV antigens. Geometric mean IgG (OD at 450 nm) and standard deviation of mpox-convalescent (“MPX”; *n* = 5), MVA-BN-vaccinated (“VAC”; *n* = 3), and controls (“CTRL”; *n* = 3).

In the preliminary screen of the 12 MPXV/VACV proteins (*n* = 5 convalescent mpox patients, *n* = 3 MVA-BN-vaccinated, and *n* = 3 uninfected/unvaccinated controls), mpox infection was associated with an increase in IgG antibody for A27, A29, A35, B16, B21, D6, E8, H3, and I1. A30 and L1 IgG were low in convalescent individuals, while C19 showed high cross-reactivity with the control sera. Vaccinated individuals showed low levels of IgG antibodies for most antigens, with E8 being a notable exception, which was also the most consistently reactive antigen in the convalescent sera (Figure 1). 

### 3.2. Assay Validation

**Figure 2:**
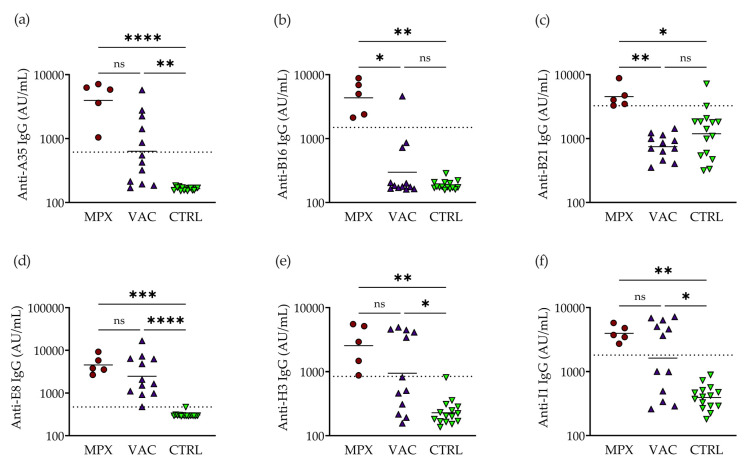
Assay validation. Level of serum IgG antibody [arbitrary units (AU)/mL] in mpox-convalescent (“MPX”; dark red circle; *n* = 5), MVA-BN-vaccinated (“VAC”; dark violet upward triangle; *n* = 12), and controls (“CTRL”; green downward triangle; *n* = 15) to MPXV proteins (**a**) A35, (**b**) B16, (**c**) B21, (**d**) E8, (**e**) H3, and (**f**) I1. Dotted lines indicate sensitivity/specificity cutoffs for (**a**,**c**,**e**,**f**) MPX vs. CTRL, (**b**) MPX vs. VAC, and (**d**) VAC vs. CTRL. ns: not significant, * *p* ≤ 0.05, ** *p* ≤ 0.01, *** *p* ≤ 0.001, **** *p* ≤ 0.0001.

We pursued validation of the A35-, B16-, B21-, E8-, H3-, and I1-specific IgG assays among the larger sample pools as these showed high reactivity with the convalescent sera. While H3 IgG showed moderate cross-reactivity with a control, we chose to pursue its validation as previous work has suggested it has utility as a marker of mpox neutralization capacity [28,33]. Among a larger sample set of *n* = 5 convalescent, *n* = 12 vaccinated, and *n* = 15 controls, we confirmed significantly increased serum IgG [arbitrary units (AU)/mL] in mpox-convalescent individuals compared to the controls for A35 (4771 [95% confidence interval (CI): 1727–7815] vs. 164.7 [159.2–170.1], *p* < 0.0001), B16 (5041 [1472–8611] vs. 187.8 [169.4–206.2], *p* = 0.0019), B21 (4859 [2047–7670] vs. 1703 [750.1–2656], *p* = 0.0242), E8 (4998 [1742–8254] vs. 307.7 [282.4–333.0], *p* = 0.0006), H3 (3185 [579.2–5791] vs. 256.4 [164.0–348.8], *p* = 0.0017), and I1 (4086 [2608–5564] vs. 428.6 [322.5–534.7], *p* = 0.0074). The background was generally low apart from for B21, for which the control sera showed high reactivity. The serum from the vaccinated individuals showed significantly increased IgG compared to the controls for A35 (1255 [204.4–2306] vs. 164.7 [159.2–170.1], *p* < 0.0011); E8 (4156 [1219–7093] vs. 307.7 [282.4–333.0], *p* < 0.0001); H3 (2008 [706.0–3311 vs. 256.4 [164.0–348.8], *p* = 0.0237); and I1 (3079 [1298–4860] vs. 428.6 [322.5–534.7], *p* = 0.0210) (Figure 2). E8 IgG showed 100% [95% CI: 75.75–100] sensitivity and 100% [79.61–100] specificity for detecting and distinguishing vaccinated individuals from the controls (Figure 2d). Compared to after vaccination, both B16 IgG (5041 [1472–8611] vs. 647.5 [0–1452], *p* = 0.0128) and B21 IgG (4859 [2047–7670] vs. 815 [600.0–1030], *p* = 0.0011) were significantly higher after infection. B16 IgG showed high sensitivity (100% [56.55–100.0%]) and specificity (91.67% [64.61–99.57%]) for detecting and distinguishing infection from MVA-BN vaccination (Figure 2b).

### 3.3. Antibody Seroprevalence

The population antibody seroprevalence was conducted using five MPXV antigen-based assays (A35, B16, E8, H3, and I1); B21 was not used for seroprevalence due to its high background. In the results, 39/214 (18.2%) of the recent samples and 5/32 (15.6%) of the controls were positive for IgG specific to ≥1 antigen, while 28/214 (13.1%) were positive for IgG specific to a single antigen. (Table 2).

**Figure 3:**
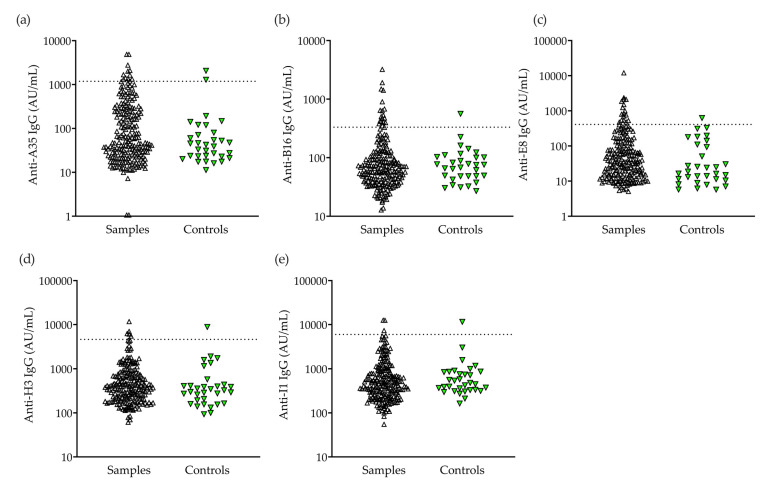
Antibody seroprevalence. Level of IgG antibody [arbitrary units (AU)/mL] to mpox proteins (**a**) A35, (**b**) B16, (**c**) H3, **(d**) I1, and (**e**) E8 in serum samples collected between June 2022 and January 2023 (black upward triangle; *n* = 214) and young pre-pandemic controls (green downward triangle; *n* = 32). Dotted lines indicate an antibody positivity cutoff of two and a half standard deviations above the control mean.

**Figure 4:**
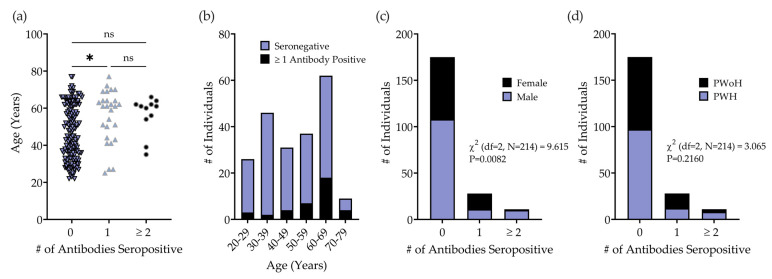
Association of MPXV antibody positivity with demographic data. (**a**) Scattered dot plot showing the age of individuals with 0 (dark blue downward triangle; *n =* 175), 1 (light blue upward triangle; *n* = 28), or ≥2 (black circle, *n* = 11) antibodies seropositive. (**b**) Distribution of age by decade of the 214 individuals included in the seroprevalence study. Distribution of (**c**) gender and (**d**) HIV status based on MPXV antibody seropositivity status. ns: not significant, * *p* ≤ 0.05.

B16 IgG positivity was most common and was observed among 18/214 (8.4%) samples and 1/32 (3.1%) controls (Figure 3). Further, 175/214 (81.8%) individuals were negative for all tested antibodies, including 92/122 (75.4%) individuals born before 1980. Individuals seropositive for a single antibody were significantly older than individuals with negative serology (55.6 [95% CI: 50.0–61.3] vs. 47.2 [45.0–49.4], *p* = 0.0234). (Figure 3a). The antibody positivity rate showed a linear trend for decade of age, being lowest among individuals aged 30–39 years old (4.3%), and highest among individuals aged 70–79 years old (44.4%) (Figure 4b). IgG positivity to ≥2 MPXV antigens, the predefined criterion for mpox seropositivity, was observed among 11/214 (5.1%) recently collected samples and 1/32 (3.1%) of the controls. Age was not significantly greater in the mpox-seropositive (≥2 antibodies seropositive) group compared to the seronegative group (56.3 [49.5–63.2] vs. 47.2 [45.0–49.4], *p* = 0.2287) (Figure 4a; Table 2). Two individuals seropositive for ≥3 MPXV antibodies, both male PWH, were born after 1980. Antibody positivity was associated with self-identified gender, with 10/11 (91.0%) mpox-seropositive, 11/28 (39.3%) single-antibody-positive, and 108/175 (61.7%) seronegative individuals being male [X^2^ (df = 2, *n* = 214) = 9.615, *p* = 0.0082] (Figure 4c). The overall mpox seropositivity was 10/129 (7.8%) among males and 1/85 (1.2%) among females (*p* = 0.0532). A two-fold higher prevalence of seropositivity was observed among PWH compared to PWoH [8/117 (6.8%) vs. 3/97 (3.1%)]; however, the association between HIV status and antibody positivity was not statistically significant [X^2^ (df = 2, *n* = 214) = 3.065, *p* = 0.2160] (Figure 4d). Among mpox-seropositive individuals, A35 positivity was most common (9/11), followed by B16 (8/11) and E8 (6/10), while I1 positivity was not observed among any of these individuals (Table 3).

## 4. Discussion

Serological analysis has utility in the identification of individual- and population-level immunity to multiple infectious agents, yet has scarcely been utilized during the ongoing mpox outbreak. VACV particle-based immunoassays, often used for OPXV serology, are logistically complicated and cannot distinguish prior mpox infection from recent or distant vaccination [34]. We evaluated the humoral immune response to multiple MPXV and VACV antigens following mpox infection or MVA-BN vaccination and validated a set of seroassays which detect mpox- and vaccine-induced IgG. B16 IgG was highly sensitive (100%) for detecting mpox infection and able to distinguish mpox-infected from MVA-BN-vaccinated individuals with 91.7% specificity. Why infection but not vaccination with MVA-BN elicited a strong response to B16, a secreted protein which is highly homologous to VACV B19 [35], remains to be determined. IgG to B21, a protein absent in VACV, was higher in convalescent than in vaccinated individuals [26]. However, due to its high reactivity among the control sera, the full-length B21 antigen is not optimal for serological assays. Mpox infection was associated with a broader and stronger humoral immune response compared to MVA-BN vaccination, in agreement with recent studies [36]. MVA-BN-vaccinated individuals had significantly increased IgG antibodies for A35, E8, H3, and I1, although more heterogeneity was seen than in the convalescent individuals or controls. This increased heterogeneity could reflect differences in vaccine administration route or dose (see discussion below). While mpox infection appears to drive a consistently robust humoral immune response to these antigens, exposure to a lower antigen dose from vaccination with a replication-deficient MVA-BN vaccine may limit the response to immunodominant epitopes such as E8, which was found consistently among vaccinated individuals. In the context of lower antigen exposure, individual differences in immunocompetence may further exacerbate the variability in humoral immune responses. 

We identified E8 IgG as a marker of MVA-BN-vaccine-induced immunity present in all MVA-BN-vaccinated PWH and PWoH. E8 is a MPXV surface-binding protein essential for viral attachment to host cells which has proven highly immunogenic and protective against VACV challenge in an animal vaccine model [37,38]. Consequently, E8 IgG induction may partly underly the protective effect of MVA-BN vaccination, which otherwise produces low or variable levels of MPXV-specific humoral immunity. Given the high levels of E8 IgG measured after mpox infection and vaccination, and its low background in the controls, E8 is highly useful for conducting OPXV serology screening and may offer an alternative to VACV-particle-based assays. Future studies are needed to correlate post-vaccination E8 titers with the risk of mpox disease in humans, which could validate the use of E8 IgG as a marker of protection. Studies are also needed to assess the durability of MPXV antigen-specific titers to evaluate the longevity of each antigen-specific response. 

Using five antigen-based assays, we found an 18.2% prevalence of MPXV antibodies among a population of 214 individuals composed largely of PWH, excluding individuals who self-reported mpox vaccination or infection. One or two controls were positive in each of the five assays, suggesting a level of non-specificity which may result from cross-reactivity with homologs of human proteins. Conversely, only one pre-pandemic control exhibited seropositivity for two or more antibodies, reaffirming the importance of using multiple antigen-based assays for serological analysis. We identified 11/214 (5.1%) samples collected during the current outbreak which were seropositive for two or more antibodies. Seropositivity for one, but not multiple, MXPV antibodies was positively correlated with age. Seropositivity for multiple antibodies may therefore reflect prior mpox infection, which is most common among younger individuals [39], while older individuals born during the era of routine childhood smallpox vaccination comprised a greater proportion of the single-antibody-positive group. In order to address the confounding effects of age, gender, and HIV status on antibody responses, we performed additional multinomial logistic regression analysis and found no significant association between seropositivity to two or more antibodies and age or HIV status (Supplementary Table S1). We identified two young individuals with no history of mpox infection or MVA-BN vaccination positive for ≥3 MPXV antibodies who may represent asymptomatic infections. We found the highest frequency of seropositivity to A35 (9/11) and B16 (8/11) among mpox-seropositive individuals. Further longitudinal evaluations of these responses could reveal whether A35 and B16 IgG are more durable following mpox infection, which could produce higher seropositivity at the time of conducting the serosurvey. While the prevalence of MPXV antibodies found here far exceeds the prevalence of reported mpox cases in the population, our findings suggest that the vast majority (>80%) of the studied population remains naïve to MPXV, with 81.8% of individuals demonstrating negative serology [40]. Seroprevalence studies conducted prior to the global mpox outbreak have found similarly low rates of anti-OPXV seropositivity, including among individuals born in the era of routine smallpox vaccination [41]. We found a high seroprevalence (6.8%) of mpox seropositivity in PWH, who have been disproportionately affected by mpox [42]. Of the mpox-seropositive individuals, 10/11 (91%) were male, in line with epidemiological data showing >90% of cases occur in men [8,43]. We found a significant association between antibody positivity status (0, 1, or ≥2 antibodies) and self-identified gender, while mpox seropositivity (≥2 antibodies positive) showed a trend toward increased prevalence among males compared to females [10/129 (7.8%) vs. 1/85 (1.2%), *p* = 0.053]. Strengthening global surveillance has been identified as a key area of action for preventing and containing future mpox outbreaks [44]. Our findings suggest that easily accessible and low-cost antigen-based serology could play an important role in future surveillance campaigns and identify A35, B16, and E8 assays as particularly promising targets for further validation. 

Our study presents several limitations. Notably, the number of mpox-convalescent individuals was small; as such, validation among larger groups of convalescent sera is needed to robustly determine the sensitivity and specificity of the antigen-based assays. While MVA-BN vaccination is offered at a standard subcutaneous dose or intradermally using a dose-sparing strategy, we did not collect data on the vaccine administration route. Seropositivity among older participants is likely in part explained by routine childhood smallpox vaccination. Lack of validation among long-term vaccination samples and individuals vaccinated with historical smallpox vaccines (e.g., ACAM-2000 and Dryvax) makes it difficult to fully evaluate the spectrum of MPXV/VACV immunity in the older population. Recent work suggests reactivity to some MPXV antigens among individuals who received historical smallpox vaccines [45]. However, we found MPXV antibodies in several young individuals (born after 1980), including two individuals seropositive for multiple antibodies, indicating the possibility of asymptomatic or subclinical infection in the high-risk population. Furthermore, no significant association was found between age and seropositivity for multiple antibodies, while the association with self-identified male gender agrees with the epidemiological data of the global outbreak rather than routine childhood vaccination. As we utilized a stringent cutoff (two and a half standard deviations above the negative control mean) to define antibody positivity to minimize background, the true seropositivity rates may exceed those found in the present study. As seroprevalence was conducted in a population with high reported rates of mpox disease (PWH in South Florida), these data may not necessarily extrapolate to different demographic or geographic contexts, including regions where HIV prevalence is low.

Finally, as participant data were self-reported, individuals may have omitted disclosing history of mpox vaccination or infection; as such, mpox seropositivity cannot conclusively represent asymptomatic infection. Further assay validation is necessary among larger cohorts of mpox-convalescent individuals and among individuals who received childhood smallpox vaccinations. Longitudinal studies may reveal the scale and temporality of the antibody response to various OPXV antigens and validate the use of these assays at specific post-vaccination and convalescent timepoints. Further studies are warranted to understand asymptomatic/subclinical infection in the context of pre-existing immunity or ongoing disease transmission. Collectively, the results presented in this study support the use of antigen-based assays for the detection of immunity to mpox infection and vaccination in large populations, including in PWH.

## Figures and Tables

**Table 1:** Demographic data of mpox-convalescent and MVA-BN-vaccinated participants.

ID	Age	Gender	HIV Status	MVA-BN Dose 1 Date	MVA-BN Dose 2 Date	Mpox Infection (Date Confirmed)	Serum Collection Date
V1	57	Male	Pos	4 October 2022	··	··	18 October 2022
V2	40	Male	Pos	30 August 2022	6 October 2022	··	6 October 2022
V3	58	Female	Pos	7 September 2022	5 October 2022	··	31 October 2022
V4	52	Male	Pos	4 October 2022	··	··	31 October 2022
V5	54	Male	Pos	5 September 2022	5 October 2022	··	28 October 2022
V6	57	Male	Pos	4 October 2022	4 November 2022	··	23 November 2022
V7	32	Female	Neg	1 July 2022	··	··	10 November 2022
V8	61	Male	Pos	15 October 2022	··	··	12 December 2022
V9	65	Male	Pos	1 September 2022	··	··	5 January 2023
V10	66	Male	Pos	21 September 2022	··	··	29 December 2022
V11	24	Male	Neg	29 July 2022	26 August 2022	··	6 September 2023
V12	23	Male	Neg	13 August 2022	13 September 2022	··	23 September 2022
M1	28	Male	Neg	22 June 2022	··	10 July 2022	16 September 2022
M2	39	Male	Neg	··	··	5 August 2022	16 August 2022
M3	35	Male	Neg	··	··	21 July 2022	10 August 2022
M4	28	Male	Neg	··	··	25 July 2022	6 September 2022
M5	31	Male	Neg	··	··	6 July 2022	28 July 2022

Neg = negative, Pos = positive, V = MVA-BN-vaccinated, M = mpox-convalescent.

**Table 2:** Antibody serosurvey results.

# of Antibodies Seropositive	Total *n* (%)	Age in Years (Mean ± SD)	*n* Male (%)	*n* PWH (%)
0	175 (81.8)	47.2 ± 14.6	108 (61.7)	97 (55.4)
1	28 (13.1)	55.6 ± 14.3	11 (39.3)	12 (42.9)
≥2	11 (5.1)	56.3 ± 10.2	10 (91.0)	8 (72.7)
All Samples	214 (100%)	48.7 ± 14.7	129 (60.3)	117 (54.7)

**Table 3:** Demographic data and antibody response of mpox-seropositive samples.

	Demographics			Antibody (IgG) Positive/Negative (+/−)				
ID	Age	Gender	HIV Status	A35	B16	E8	H3	I1
S + 1	56	Male	Neg	+	−	−	+	−
S + 2	60	Female	Pos	+	−	+	−	−
S + 3	54	Male	Pos	+	+	+	−	−
S + 4	35	Male	Pos	+	+	+	+	−
S + 5	61	Male	Pos	+	+	+	−	−
S + 6	62	Male	Neg	+	−	−	+	−
S + 7	64	Male	Pos	+	+	−	−	−
S + 8	39	Male	Pos	+	+	+	−	−
S + 9	66	Male	Pos	+	+	−	−	−
S + 10	62	Male	Neg	−	+	+	−	−
S + 11	61	Male	Pos	−	+	−	+	−

Pos = positive, Neg = negative.

## Data Availability

All data that underlie the results reported in this article are available within the article and its Supplementary Materials. Additional shareable documents include the statistical analyses. Proposals should be directed to Suresh Pallikkuth (spallikkuth@med.miami.edu).

## Supplementary Materials

The following supporting information can be downloaded at https://www.mdpi.com/article/10.3390/pathogens12111355/s1: File S1: Seroprevalence sample demographic information; File S2: ELISA data. Table S1: Multinomial logistic regression analysis.
