# Supplementary material for: Serosurvey of Immunity to Monkeypox (Mpox) Virus Antigens in People Living with HIV in South Florida
Source: Pathogens. 2023 Nov 15;12(11):1355. doi: 10.3390/pathogens12111355 (PMC10675141; doi:10.3390/pathogens12111355)
Supplement: Supplementary file 1 [file pathogens-12-01355-s001.zip › Supplementary Table S1_Pathogens.pdf]

Multinomial logistic regression results:

MPXV Antibody Positivity	Intercept	HIV Status (HIV Positive)	Age	Male Gender
= 1 antibody positive	-3.374625	-0.5882291	0.04643884	-1.138332
≥2 antibodies positive	-6.765861	0.6802219	0.04242784	1.742656

P-values for variables:

MPXV Antibody Positivity	Intercept	HIV Status (HIV Positive)	Age	Male Gender
= 1 antibody positive	0.0001647653	0.1785819	0.003593848	0.01043478
≥2 antibodies positive	0.0001193892	0.3354981	0.089172560	0.10321792
